# Establishing an ad hoc COVID-19 mortality surveillance during the first epidemic wave in Belgium, 1 March to 21 June 2020

**DOI:** 10.2807/1560-7917.ES.2021.26.48.2001402

**Published:** 2021-12-02

**Authors:** Françoise Renard, Aline Scohy, Johan Van der Heyden, Ilse Peeters, Sara Dequeker, Eline Vandael, Nina Van Goethem, Dominique Dubourg, Louise De Viron, Anne Kongs, Naïma Hammami, Brecht Devleesschauwer, André Sasse, Javiera Rebolledo Gonzalez, Natalia Bustos Sierra

**Affiliations:** 1 Department of Epidemiology and Public Health, Sciensano, Brussels, Belgium; 2 Agence pour une Vie de Qualité (AViQ), Charleroi, Belgium; 3 Commission Communautaire Commune de la Région de Bruxelles-Capitale (COCOM), Brussels, Belgium; 4 Agentschap Zorg en Gezondheid (AZG), Vlaanderen, Brussels, Belgium; 5 Department of Veterinary Public Health and Food Safety, Ghent University, Merelbeke, Belgium

**Keywords:** mortality, COVID-19, surveillance, Belgium

## Abstract

**Background:**

COVID-19-related mortality in Belgium has drawn attention for two reasons: its high level, and a good completeness in reporting of deaths. An ad hoc surveillance was established to register COVID-19 death numbers in hospitals, long-term care facilities (LTCF) and the community. Belgium adopted broad inclusion criteria for the COVID-19 death notifications, also including possible cases, resulting in a robust correlation between COVID-19 and all-cause mortality.

**Aim:**

To document and assess the COVID-19 mortality surveillance in Belgium.

**Methods:**

We described the content and data flows of the registration and we assessed the situation as of 21 June 2020, 103 days after the first death attributable to COVID-19 in Belgium. We calculated the participation rate, the notification delay, the percentage of error detected, and the results of additional investigations.

**Results:**

The participation rate was 100% for hospitals and 83% for nursing homes. Of all deaths, 85% were recorded within 2 calendar days: 11% within the same day, 41% after 1 day and 33% after 2 days, with a quicker notification in hospitals than in LTCF. Corrections of detected errors reduced the death toll by 5%.

**Conclusion:**

Belgium implemented a rather complete surveillance of COVID-19 mortality, on account of a rapid investment of the hospitals and LTCF. LTCF could build on past experience of previous surveys and surveillance activities. The adoption of an extended definition of ‘COVID-19-related deaths’ in a context of limited testing capacity has provided timely information about the severity of the epidemic.

## Introduction

The new coronavirus disease (COVID-19), first described in December 2019 in Wuhan, China [1], was detected for the first time in Belgium on 4 February 2020 in an asymptomatic person who tested positive for severe acute respiratory syndrome coronavirus 2 (SARS-CoV-2) [2]. The first epidemic wave in Belgium formally started on 1 March 2020, marked by a rapidly increasing number of people testing positive for SARS-CoV-2 [3]. The first death occurred on 10 March. The COVID-19-related mortality in Belgium promptly drew attention for two reasons: a high COVID-19 mortality rate, and a good completeness in reporting of deaths. 

Belgium experienced a heavy death toll during the first epidemic wave (1 March–21 June 2020), with 9,712 deaths attributed to COVID-19 by day 103, representing 845 COVID-19 deaths per million inhabitants (DPM). This rate was among the highest in Europe, followed by the United Kingdom, Spain, and Italy [4]. However, differences in reporting practices have challenged international comparisons of COVID-19 mortality [5-8]. Belgium also attracted attention for its completeness of COVID-19 death reporting in the early stage of the epidemic, including all deaths potentially attributable to COVID-19, irrespective of the diagnostic method and setting. This approach was initially met with criticism, both at the national and the international levels. However, by the end of April, international comparisons revealed that Belgium had a particularly exhaustive approach of reporting COVID-19 deaths [6,9,10]. Moreover, an excellent correlation between the all-cause excess mortality and the reported COVID-19 deaths could be shown on a daily basis [11-15].

During a severe pandemic, it is critical to establish a robust surveillance to guide control measures. Mortality is a key indicator for the surveillance of the COVID-19 pandemic [16], by informing about the epidemic’s severity. However, the standard registration procedure of the specific causes of death through death certificates is typically a 2-year process in Belgium [17]. For this reason, an ad hoc surveillance of COVID-19 deaths was established to respond to this public health emergency. 

Here we describe and assess the surveillance of COVID-19-related mortality in Belgium during the first epidemic wave, and aim to draw lessons from this experience.

## Methods

### Implementation of COVID-19 mortality surveillance in Belgium

#### Initiation of COVID-19 mortality surveillance

In Belgium, recommendations for measures to control unexpected public health threats are usually developed by the Risk Assessment Group (RAG) – composed of representatives of all health administrations and experts – and submitted to the Risk Management Group (RMG) – composed of representatives of the Ministers of Health – that takes decisions [18,19]. By the end of January 2020, COVID-19 was placed on the list of mandatory notifiable infectious diseases, initially in a generic category of ‘infectious problem with a particular or unusual presentation’ [20] and after a few weeks, in a SARS-specific category, obliging health professionals to report any case of this disease to the competent regional health authorities (RegHAs). In early March 2020, a COVID-19 surveillance plan was developed by the regional and federal health authorities together with the Department of Epidemiology and Public Health of Sciensano, the Belgian institute for health [21].

#### COVID-19 case classification and death notification

Case definitions evolved during the first epidemic wave. Changes occurred in parallel to the overall knowledge of the disease and with the progressive inclusion of laboratory and, subsequently, radiological criteria (Supplementary Table S1) [22]. From May 2020 onwards, the European Centre for Disease Prevention and Control (ECDC) defined a ‘confirmed’ COVID-19 case as meeting the laboratory criteria, a ‘probable’ case as meeting the clinical criteria with an epidemiological link or with radiological evidence showing lesions compatible with COVID-19, and a ‘possible’ case as meeting the sole clinical criteria (Table 1) [23]. Belgium used a slightly different case classification, with ‘confirmed’ and ‘possible’ categories identical to the ECDC categories, but without using ECDC’s ‘probable’ category (Table 1). A ‘radiologically confirmed’ category was created, defined as a compatible chest computed tomography (CT) scan in a person with suggestive clinical presentation and a negative laboratory test [24-26].

**Table 1:** Criteria, classifications and definitions for cases and deaths attributable to COVID-19, European Centre for Disease Prevention and Control and Belgium, March–June 2020

**Criteria**	**ECDC**	**Belgium**
	**5 May 2020^a^**	**15 May 2020^a^**
Laboratory	Detection of SARS-CoV-2 nucleic acid in a clinical specimen	SARS-CoV-2 infection confirmed by a molecular test
Epidemiological	Epidemiological link with a confirmed COVID-19 case	Not used in Belgium
Diagnostic imaging	Radiological evidence showing lesions compatible with COVID-19	Chest CT scan showing lesions compatible with COVID-19
Clinical	At least one of the following symptoms: cough, fever, shortness of breath, sudden onset of anosmia, ageusia or dysgeusia	At least one of the following symptoms that appear with no other obvious cause: cough, dyspnoea, thoracic pain, acute anosmia or dysgeusia OR at least two of the following symptoms with no other obvious cause: fever, muscle pain, fatigue, rhinitis, sore throat, headache, anorexia, watery diarrhoea, acute confusion, sudden fall OR deterioration if the patient shows chronic respiratory symptoms
COVID-19 case classification	ECDC	Belgium
	5 May 2020	15 May 2020
Confirmed case	A person meeting the laboratory criteria	A person meeting the laboratory criteria
Probable case	Any person meeting the clinical criteria AND epidemiological criteria OR any person meeting diagnostic imaging criteria	Not used in Belgium
Radiologically-confirmed case	Does not exist	Suggestive clinical presentation AND a compatible chest CT scan AND the RT-PCR for COVID-19 is negative
Possible case	Any person meeting the clinical criteria	Any person meeting the clinical criteria
COVID-19 death definition^b^	ECDC–WHO	Belgium
	20 Apr 2020	30 Mar 2020
COVID-19-related death	Death resulting from a clinically-compatible illness in a probable or confirmed COVID-19 case, unless a clear alternative cause of death unrelated to COVID-19 is identified	Death in a confirmed, radiologically-confirmed or possible case that occurred in any setting, unless a clear alternative cause of death unrelated to COVID-19 is identified

COVID-19: coronavirus disease; CT: computed tomography; ECDC: European Centre for Disease Prevention and Control; SARS-CoV-2: severe acute respiratory syndrome coronavirus 2; WHO: World Health Organization.

^a^ The date of the last definition that was enacted during the period we refer to (up to 21 June 2020).

^b^ The date of adaptation of the definition of a COVID-19 death. In Belgium, the definition was retrospectively applied to previous deaths when the information was available.

For the surveillance of deaths, the World Health Organization (WHO) [27], and later ECDC [23], created a definition for deaths attributable to COVID-19 for surveillance purposes as ‘deaths resulting from a clinically compatible illness, in a probable or confirmed COVID-19 case, unless there is a clear alternative cause of death that cannot be related to COVID-19 disease, e.g. trauma’ [28]. Belgium adopted broader inclusion criteria for notifying deaths attributable to COVID-19 by reporting deaths in ‘possible’ cases. The rationale for including these cases was the very low testing capacity during the first weeks of the epidemic (on account of a shortage of reagents and swabs), leading to a quasi-impossibility to get a laboratory-confirmed diagnosis outside hospitals. The criteria were first broadened to deaths of possible cases in LTCF (from 30 March), followed by in-hospital deaths (from 5 May). At the same time, deaths of ‘radiologically-confirmed’ cases were included in the case definitions, after the recognition of the added value of a chest CT scan as a diagnostic tool [25,26]. The new inclusion criteria were also applied retrospectively.

#### Health authorities and settings involved in COVID-19 surveillance

The modalities of the surveillance differed based on the setting (hospital, LTCF and the community) and according to the health authorities involved. Belgium is a federal state composed of regions and communities [29]. For the COVID-19 surveillance, responsibilities for data collection in hospitals, LTCF and the community were shared between the federal state and four federated entities [30], namely the Flemish Region (Flanders), the Brussels-Capital Region (Brussels), the Walloon Region (Wallonia), and the German-speaking Community (GSC). The respective RegHAs are the Agentschap Zorg en Gezondheid (AZG) for Flanders, Agence pour une Vie de Qualité (AViQ) for Wallonia, the Commission Communautaire Commune de la Région de Bruxelles-Capitale (COCOM) for Brussels, and the Ministry of the GSC.

The Belgian Federal Public Service Public Health, Food Chain Safety and Environment (SPF-PH) is responsible for the organisation of the 103 acute hospitals and 60 psychiatric hospitals [31]; the SPF-PH delegated the COVID-19 data collection of acute hospitals directly to Sciensano, while psychiatric hospitals had to notify COVID-19 cases and deaths to the RegHAs.

**Figure 1:**
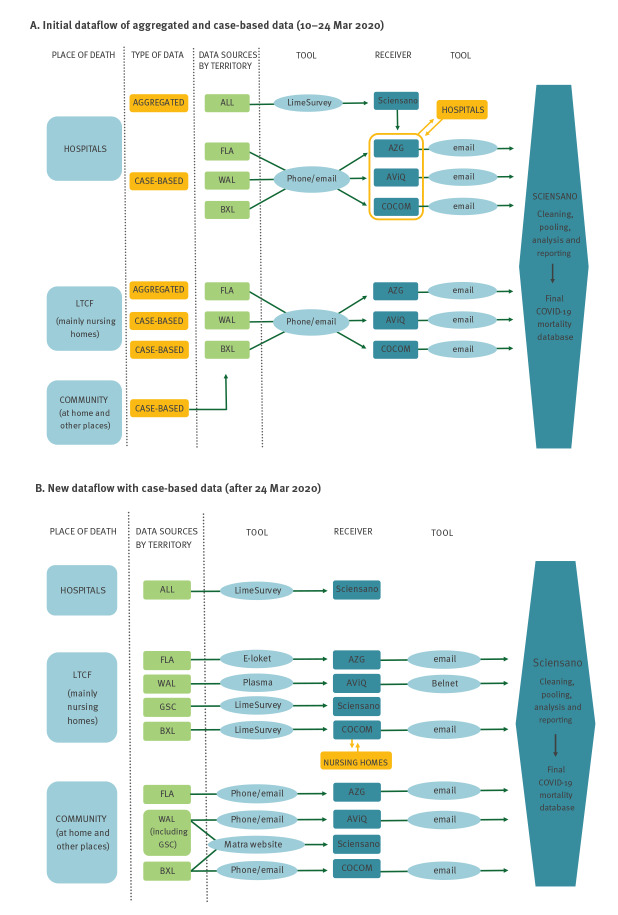
Data flow of COVID-19 mortality surveillance in the first epidemic wave, Belgium, 10 March–21 June 2020

AViQ: Agence pour une Vie de qualité; AZG: Agentschap Zorg en Gezondheid; BXL: Brussels-Capital Region; COCOM: Commission Communautaire Commune de la Région de Bruxelles-Capitale; COVID-19: coronavirus disease; FLA: Flanders; GSC: German-speaking Community; LTCF: long-term care facilities; WAL: Wallonia.

The RegHAs are responsible for the officially accredited LTCF [32], including 1,542 nursing homes (NH) and around 1,000 residential services for elderly people, 59 psychiatric care facilities, institutions for people with disabilities and revalidation centres (exact number currently unknown). The RegHAs organised the data collection in LTCF, before transmitting the data to Sciensano (Figure 1). COVID-19-related deaths in the community were also reported by medical doctors (MDs) to the RegHAs.

Panel A: Aggregated and case-based data. The yellow lines represent the double flow of information between hospitals and RegHAs. Hospitals notified case-based data spontaneously to RegHAs. Sciensano asked the RegHAs to contact hospitals to obtain missing case-based data when these had been reported in an aggregated way in the surge capacity survey, but not yet individually to the RegHAs.

Panel B: Case-based data. The yellow line represents the exchanges between COCOM and the nursing homes for data quality before sending the data to Sciensano.

Tools: The data platforms used were different for each territory. Belnet FileSender: national platform for secure data transmission (https://www.belnet.be/en); e-loket: specific online tool for AZG; LimeSurvey: (https://www.limesurvey.org); Matra website: specific online tool from Sciensano; Plasma: specific online tool for AViQ.

#### Data sources and data flows

The data sources for the COVID-19 deaths included hospitals, LTCF and MDs for deaths that occurred in the community.

In the first weeks of the epidemic, COVID-19 cases and deaths in any setting were notified by MDs who then certified the deaths to the health inspectors of the respective RegHAs. This data flow had been the long-standing official procedure in Belgium for reporting cases of notifiable infectious diseases.

On 17 March 2020, Sciensano initiated a centralised data registration by gathering information on deaths from the RegHAs (initially case-based), and from hospitals (initially aggregated data). The difference between individual and aggregated data is explained further, under the heading ‘Specification of the reported information’. 

##### Surveillance in hospitals

Within the framework of the newly developed ‘Hospital and Transport Surge Capacity’ plan [33], a single data collection – the surge capacity (SC) survey – was implemented in all general hospitals in Belgium on 10 March 2020; the flow became fully operational on 15 March [34]. Hospitals were tasked to register COVID-19 admissions, stays, intensive care unit occupation, discharges and deaths on an aggregated basis via an online questionnaire developed in LimeSurvey by Sciensano. This surveillance did not include psychiatric hospitals for which the deaths were declared to the RegHAs. A double flow of information about in-hospital deaths coexisted for two weeks (Figure 1A), as hospitals had to report both COVID-19 deaths aggregated through the SC survey and also case-based deaths to the RegHAs. In addition, Sciensano sent the daily number of deaths from each hospital extracted from the SC survey to the respective health inspectors, in order to complete missing information when necessary.

Given the rapid increase in the number of cases and deaths, the workload quickly overwhelmed both the hospitals, which strained to care for patients with an added administrative burden of reporting each death twice, and the health inspectors, who prioritised supporting the field work. At this point, it was evident that the initial official data flow was unsustainable. On 24 March 2020, the SC survey was modified to allow the online registration of case-based individual death data, which then became the official source of information for hospital deaths (Figure 1B). Thus, hospital information was provided directly to Sciensano, which reduced the workload of hospitals and health inspectors. Since participation in the SC survey was mandatory [35], the hospital mortality data accurately reflected the situation in hospitals.

##### Surveillance in long-term care facilities

A COVID-19 case and death surveillance was promptly implemented in LTCF by the RegHAs at the outset of the epidemic. Surveillance in LTCF was initially focused on the NH, where most cases and deaths occurred. The RegHAs sent their data to Sciensano on a daily basis (Figure 1). The RegHAs set up online data collection systems and urged the NH to participate. However, each RegHA used slightly different questionnaires and recording tools, resulting in tedious work for Sciensano to pool the data. The chronology of the events and details about the tools are described in Supplementary Table S2. For the LTCF other than NH, the organisation of the surveillance of cases and deaths is still a work in progress. At the time of writing, the data in non-NH LTCF was still being consolidated. Therefore, the participation rate in non-NH LTCF could not be calculated, and the death registration may be incomplete in those settings. 

##### Surveillance in the community

The modalities of reporting deaths in the community (at home or other places outside hospitals and LTCF) also differed across regions. In Flanders and Brussels, deaths that occurred at home or in other settings were first notified by MDs to the RegHAs, and then to Sciensano. In Wallonia and GSC, MDs notified deaths to RegHAs and directly to Sciensano (since 9 April) via the online tool ’Matra’ (some deaths in Brussels have also been notified via Matra). Deaths of NH residents who returned home to their family for their final moments were notified via the NH online tool as ‘death at home’. However, very few cases were notified through those data flows (< 1% of all COVID-19 deaths). 

The data flow of all COVID-19 deaths results in the transmission of a total of nine data files (Figure 1B).

#### Specification of the reported information 

The data description of the variables and its evolution is provided in Supplementary Table S3. The variables that were collected included sex, age, date of death, place of occurrence, case classification, and residence in a LTCF.

The format of the data transmitted to Sciensano on COVID-19 deaths changed over time, with some data collections initially consisting of aggregated data broken down by institution and date, but not by age and sex. In hospitals, the date of death was approximated by the notification date, whereas in LTCF, the notification date minus one day was used as a best proxy, since data were transmitted in the morning. Settings in all regions evolved towards a case-based data transmission (Supplementary Table S3) and details about initially aggregated data could be retrospectively retrieved.

COVID-19 death data collected in hospitals were initially transmitted in an aggregated format until 23 March, and thereafter, data were case-based. The dictionary of the variables and the timeline of their introduction is shown in Supplementary Table S3.

In LTCF, online data collection on deaths was case-based from the start in Wallonia. In Brussels, online data were transmitted in an aggregated way until 11 April, but the COCOM contacted LTCF to obtain individual details. Similarly, aggregated data from LTCF were initially sent by the GSC until 12 May and the Flemish Region until 1 June, after which case-based data were sent. In some regions, data were completed retrospectively when available. For the GSC, details of previously transmitted aggregated deaths were retrospectively searched for by Sciensano in July 2020; AZG conducted a retrospective case-based inventory of all NH deaths in the summer, allowing an update of the final COVID-19 mortality database with the details of all deaths on 25 August (see section ‘Rectification of errors’). The case-based data of the inventory were considered to be more valid than the previous aggregated ones.

#### Data quality checks

The use of all online tools included data quality checks before data entry to avoid aberrant values. In the case-based data collection, LTCF could make corrections to a previously entered case, by re-entering it as an update.

Data quality checks were performed routinely to search for duplicates and aberrant, implausible and missing values, first at the level of the data providers, i.e. RegHAs and the Sciensano hospital data collection team, and then by the Sciensano mortality team. When data included sufficient individual details, some data entry errors could be identified by automated detection, e.g. obvious duplicates, while others had to be verified with the data providers. Other types of errors could only be found after an intensive search by the RegHAs, which led to late corrections. Finally, errors could also be reported spontaneously by the sources. Automated quality controls developed gradually, through experiencing new types of errors as well as discussions with the different data providers.

#### Pooling the files into the final COVID-19 mortality database

Sciensano harmonised the formats and restructured aggregated data files to match the case-based data. The data were then cleaned, and the nine data files were pooled into the final COVID-19 mortality database (Figure 1B). To avoid double registration, records of LTCF residents notified by the RegHAs as having died in the hospital were not entered in the database, as they were already notified by the hospitals.

#### Special registration events

On special occasions, a catch-up of backlogged deaths occurred, either because of changes in Sciensano’s criteria to include COVID-19 deaths or by the addition of new data flows (Table 2). On 30 March 2020, deaths of possible cases in NH and in the community were included. On 3 April, the online transmission between AZG and Sciensano regarding the NH deaths in Flanders was operationalised to include deaths in the central database on 6 April. On 21 April, Sciensano included the NH deaths of the GSC. On 5 May, deaths of radiologically-confirmed and possible cases in hospitals were included. On 23 May, the deaths registered via the Matra online tool in Wallonia, GSC and Brussels were included.

**Table 2:** Special registration events of backlogged COVID-19 deaths, Belgium, 10 March–21 June 2020 (n = 786)

Special event	Date	Hospitals	LTCF	Community	Total
Deaths of possible cases in LTCF and in the community	30 Mar	NA	64	17	81
Deaths in hospitals before 24 March, after Investigation 1^a^	3 Apr	26	1	NA	27
LTCF deaths of Flanders	6 Apr	NA	240	2	242
LTCF deaths of Flanders after Investigation 2^a^	8 Apr	NA	171	NA	171
LTCF deaths of GSC	21 Apr	NA	23	0	23
Deaths of radiologically-confirmed and possible cases in hospitals after Investigation 3^a^	5 May	232	NA	NA	232
Data flow from website Matra in Wallonia, GSC and Brussels	23 May	NA	NA	10	10
Total number of deaths	NA	258	499	29	786

COVID-19: coronavirus disease; GSC: German-speaking Community; LTCF: long-term care facilities; NA: not applicable.

^a^ Sciensano actively conducted three separate investigations (Investigations 1, 2 and 3) to recover unreported deaths or to complete information.

Additionally, Sciensano actively conducted three investigations to recover unreported deaths or to complete information. Investigation 1 concerned deaths that occurred in hospitals between 15–23 March – a period during which details of in-hospital deaths were not fully declared to the RegHAs. These deaths were sought after in an additional source, the ‘hospital clinical survey’ [34], which provided 27 COVID-19 deaths (including one death in a revalidation centre) that were introduced in the final database on 1–3 April.

Investigation 2 concerned deaths among NH residents in Flanders. Sciensano surveyed 152 NH in Flanders that reported COVID-19 deaths during the period of 18–31 March, but for which the resident’s place of death had not been initially requested (n = 333). Of those, 171 COVID-19 deaths could be included as COVID-19 deaths in NH, while 50 in-hospital deaths, already notified by the hospital flow were not added. Forty reported deaths were non-COVID-19 deaths, and 40 other cases were registration errors. Of all NH, 14 could not provide the information or could not be contacted, leaving 32 deaths possibly unaccounted for.

Investigation 3 was completed on 4 May in 60 hospitals that declared 195 deaths of possible cases that occurred before 11 April, i.e. the date on which the SC survey allowed the registration of radiologically-confirmed cases. The objective was to obtain more information before including those deaths in the COVID-19 mortality database. Among those, 169 deaths could be reclassified as either laboratory-confirmed cases (n = 25), chest CT scan-confirmed cases (n = 71), clinical cases (n = 53), or non-COVID-19 cases (n = 20), leaving 26 deaths possibly unaccounted for since the cause could not be validated; 149 deaths were included that had occurred between 10 March and 11 April. Following this investigation, Sciensano decided to include radiologically-confirmed as well as clinically-diagnosed COVID-19 deaths that occurred in hospitals in the statistics, leading to the inclusion of an additional 83 deaths for the period 12 April to 5 May. In total 232 retrospective deaths were included on 5 May.

#### Reporting and dissemination of results

The last step of the surveillance was to ensure that publication of the results related to COVID-19 deaths was executed in a way that could support public health and research. The data in the final COVID-19 mortality database were analysed daily to produce and publish official COVID-19 mortality figures.

The number of COVID-19 deaths was reported by Sciensano using two different indicators: the total number notified within the past 24 h and the number by date of occurrence. On 14 March, when four COVID-19 deaths had been reported, the first daily epidemiological report of Sciensano was published. Between 26 March and 21 June 2020, 13 additional weekly epidemiological reports were published, which included additional information on all-cause mortality, deaths in NH and case fatality rate. Daily and weekly reports can be found at https://covid-19.sciensano.be/fr/covid-19-situation-epidemiologique.

On 31 March 2020, Sciensano launched its open data platform (https://epistat.wiv-isp.be/covid), where aggregated data on COVID-19 deaths, hospitalisations, confirmed cases, and number of tested individuals were made available via stable and public URLs. The data files, available in different formats, e.g. Excel, CSV, JSON, were updated daily, and included the full time-series since the start of the outbreak. At the time of publication, the data files are still updated daily. The data are also visualised in the form of an interactive dashboard (https://datastudio.google.com/embed/reporting/c14a5cfc-cab7-4812-848c-0369173148ab/page/ZwmOB).

### Assessment of the surveillance process

An optimal assessment of the coverage and the validity of this ad hoc COVID-19 mortality surveillance set up under emergency circumstances would require the use of death certificates, which was not possible at the time. Therefore, we assessed the Belgian death surveillance process with the available information, such as participation rates, timeliness measurement, rectification of errors, reporting and dissemination.

#### Participation rates

The daily participation rate was calculated for hospitals and NH as the total number of institutions that responded on a particular day divided by the total number of institutions. Zero-reporting of cases and deaths was included for both hospitals and NH surveillance systems (except for NH in Wallonia where zero-reporting was only stated for cases). The percentage of NH that responded at least once was also calculated.

The participation rate of the other LTCF was not available during the first epidemic wave.

#### Timeliness measurement

The notification delay was calculated as the difference between the date of a death and the inclusion of this death in the final COVID-19 mortality database. Since publication occurred the day after inclusion in the database, the publication of a death had a latency of one additional day. The distribution of the notification delay (1, 2, 3–7, 8–14, 15–30, 31–60 and ˃ 60 days) was calculated for the usual data flow and for special registration events. The timeliness was measured as the percentage of deaths reported within 2 calendar days after occurrence.

#### Rectification of errors

Errors that escaped routine data-quality checks included duplicates with errors in the personal/NH identifiers, non-COVID deaths and false deaths.

The percentage of errors was calculated differently across the regions since the types of data differed. In Wallonia, Brussels and the GSC, LTCF case-based data were transmitted – or could be retrieved – in almost real-time. The RegHAs and the institutions regularly reported errors about cases included in the statistics. The percentage and cause of error notification were calculated for the whole period. In Flanders, aggregated data were initially transmitted, until 1 June. During this period, data entry checks were performed by AZG. No additional checks could be done by Sciensano on aggregated data. The case-based inventory of those deaths provided a new version of the COVID-19 deaths data, which was included on 25 August and considered as more valid. The percentage of errors was calculated by comparison between the previously transmitted aggregated numbers and the inventory.

#### Reporting and dissemination

The visibility of the reporting and dissemination steps was assessed by the number of epidemiological reports including mortality indicators and the number of times they have been downloaded.

#### Data analysis

The analysis focussed on COVID-19 deaths during the first wave of the COVID-19 epidemic, from 10 March to 21 June, extracted from the 27 August 2020 version of the final COVID-19 death database in Belgium. SAS software 9.4 (SAS Institute, Cary, North Carolina, United States (US)) was used, the graphs were performed using R version 4.1.0 (R foundation, Vienna, Austria) and Microsoft Publisher (Microsoft, Redmond, Washington, US).

### Ethical statement

Collection of aggregated data was performed within lawful grounds of the General Data Protection Regulation (GDPR). Although the GDPR does not apply to the personal data of deceased individuals, the researchers notified the Belgian Data Protection Authority. All data are saved on a secured server at Sciensano.

## Results

By the end of the first wave, 9,712 COVID-19 deaths were reported, among which 4,732 (48.7%) occurred in hospitals, 4,904 (50.5%) in LTCF, including 4,857 in NH, 32 in service flats for elderly people and 15 in other LTCF (residence care for people with disabilities or psychiatric patients, or psychiatric hospitals) and 57 (0.6%) in the community. The location of 19 deaths (0.2%) was unknown.

### Assessment of the surveillance process

#### Participation rates

After a short implementation phase, the participation rate of the 103 hospitals reached 99% on 15 March 2020 and remained very high during the whole period (Table 3).

**Table 3:** Participation rate of COVID-19 death surveillance by general hospitals and nursing homes, Belgium, 10 March–21 June 2020 (n = 1,645)

Places of death	Start online registration^a^	Start online transmission to Sciensano^a^	Total number of institutions (n = 1,645)	Participation rate (%)			
				At least once	Median	Min	Max
**Hospitals**							
** Total**	15 Mar	15 Mar	**103**	**100**	**100**	**97**	**100**
**Nursing Homes **							
Flanders	18 Mar	3 Apr	814	98	86	70	94
Wallonia	20 Mar	24 Mar	573	99	83	42	94
Brussels	26 Mar	26 Mar	147^b^	97	72	47	86
GSC	28 Mar	28 Mar	8	100	75	50	100
**Total**	NA	NA	**1,542**	**99**	**83**	**58**	**92**

COVID-19: coronavirus disease; GSC: German-speaking Community; NA: Not applicable.

^a^ All dates are in 2020.

^b^ Including eight nursing homes situated in Brussels that fall under the responsibility of Flanders.

Region-specific online tools were implemented to record deaths in NH, although the start day of recording differed slightly between the RegHAs (Supplementary Table S2); this occurred without a loss of data as the initial data flow involving health inspectors was maintained until online registration was effective. Nearly 100% of the NH reported at least once. The median daily participation rate was 83%, with a higher median participation in Flanders and Wallonia than in the other entities. Deaths not reported on a given day were usually reported at the next reporting session, leading to small notification delays but no underestimation of the numbers.

#### Timeliness measurement

In total, taking into account both the usual data flow and the special registration events, 85% of the deaths were notified within 2 calendar days, e.g. 11% of the deaths were notified the same day, 41% were reported after 1 day, and 33% of deaths were reported 2 days later (Table 4). The usual data flow resulted in 90% of deaths being recorded within 2 calendar days, e.g. 12% notified the same day, 44% after 1 day, and 34% on the second day. Notification of deaths that occurred in hospitals was faster, with 95% of deaths notified after 1 day (22% on the day of occurrence). Ca 1% of hospital deaths were notified after 8 days, because of omissions that were later corrected. Notification of deaths occurring in LTCF was slower because of data quality control steps at the RegHA level, with 2% notified the same day, 16% notified after one day, and 67% by the second day. Of the few deaths notified in the community, 60% were notified within 2 days.

**Table 4:** Latency for inclusion of COVID-19 deaths in the database for usual data flow and special registration events by place of death, Belgium, 10 March–21 June 2020 (n = 9,693)

Latency	Usual data flow								Special registration events								Total							
	Hospitals		LTCF		Community		Total		Hospitals		LTCF		Community		Total		Hospitals		LTCF		Community		Total^a^	
	n	%	n	%	n	%	n	%	n	%	n	%	n	%	n	%	n	%	n	%	n	%	n	%
Same day	1,000	22	70	2	4	14	1,074	12	1	0	0	0	1	3	2	0	1,001	21	70	1	5	9	1,076	11
1 day	3,252	73	688	16	7	25	3,947	44	12	5	11	2	2	7	25	3	3,264	69	699	14	9	16	3,972	41
2 days	122	3	2,941	67	6	21	3,069	34	2	1	87	17	0	0	89	11	124	3	3,028	62	6	11	3,158	33
3–7 days	51	1	509	12	8	29	568	6	15	6	202	40	10	34	227	29	66	1	711	14	18	32	795	8
8–14 days	5	0	116	3	0	0	121	1	41	16	151	30	5	17	197	25	46	1	267	5	5	9	318	3
15–30 days	21	0	61	1	0	0	82	1	89	34	48	10	4	14	141	18	110	2	109	2	4	7	223	2
31–60 days	15	0	20	0	3	11	38	0	98	38	0	0	7	24	105	13	113	2	20	0	10	18	143	1
> 60 days	8	0	0	0	0	0	8	0	0	0	0	0	0	0	0	0	8	0	0	0	0	0	8	0
Total	4,474	100	4,405	100	28	100	8,907	100	258^b^	100	499^c^	100	29^d^	100	786	100	4,732	100	4,904	100	57	100	9,693	100

COVID-19: coronavirus disease; LTCF: long-term care facilities.

^a^ Of the total number of deaths, 19 cases with unknown place of deaths have been excluded.

^b^ The 258 special registration deaths from hospitals include 232 deaths because of a change in the case definition of hospital deaths on 5 May and 26 deaths that were retrieved during Investigation 1 on 3 April.

^c^ The 499 special registration deaths from LTCF include 64 deaths of possible COVID-19 cases in LTCF on 30 March, one death from Investigation 1 on 3 April, 411 deaths included from Flanders during 6–8 April, and 23 deaths in LTCF in GSC on 21 April.

^d^ The 29 special registration deaths from the community include 17 deaths of possible COVID-19 cases in the community on 30 March, two deaths in the community from Flanders on 6 April, and 10 deaths from the website Matra on 23 May.

**Figure 2:**
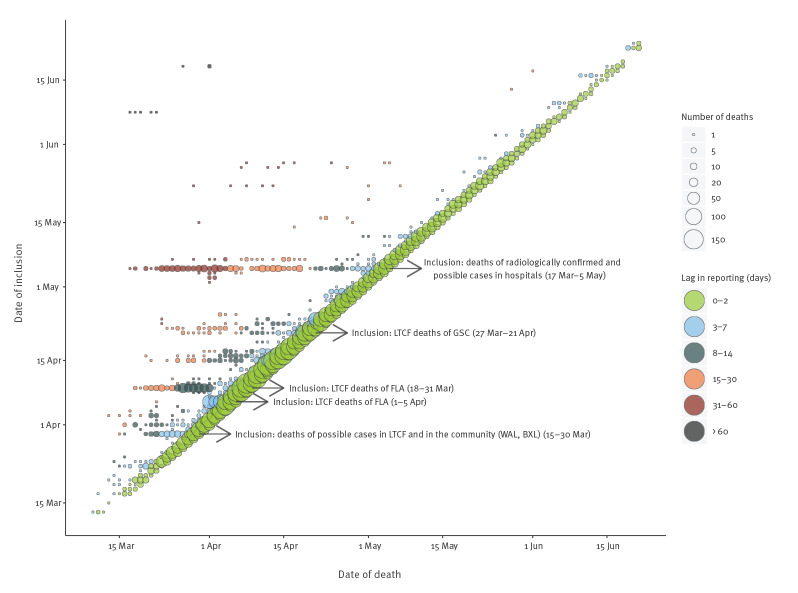
Impact of special registration events on the daily COVID-19 deaths counts, Belgium, 10 March–21 June 2020 (n = 9,712)

BXL: Brussels; COVID-19: coronavirus disease; FLA: Flanders; GSC: German-speaking Community; LTCF: long-term care facilities; WAL: Wallonia (excluding GSC).

COVID-19 deaths have also been added to the database on the occasion of ‘catch-ups’, i.e. particular events that occurred following protocol or data flow changes. Of note, seven special registration events contributed to some of the long delays observed in recording (Table 2); the length of the delay depended on the lag time between the registration of catch-ups and the date of death. The catch-up of backlogged deaths (Table 4) concerned 8% of all COVID-19 deaths (n = 786). Those catch-ups led to updates of the historical time series of the number of COVID-19 deaths. Changes in the inclusion criteria brought 313 additional deaths (at 30 March and 5 May). Delayed inclusion of data flows that had begun registration earlier revealed 473 unregistered deaths; the first online data from NH in Flanders were transmitted to Sciensano with a delay of 2 weeks, which had an important impact on the number of newly published deaths by Sciensano at that moment. Of note, peaks of backlogged deaths were observed on four particular dates: 30 March, 6 April, 8 April and 5 May (Table 2 and Figure 2). As those backlogged deaths occurred across a large time span, the change in the number of deaths by date of occurrence was much more gradual than the change of the number of deaths by registration date.

#### Rectification of errors

In the data obtained from Wallonia, Brussels and the GSC, 370 COVID-19 deaths were excluded after their publication (3.8% of the total deaths, including 17% of the LTCF deaths in those entities). Errors were either duplicates discovered after reporting (n = 148), non-COVID-19 deaths notified as COVID-19 deaths (n = 123), and data-entry errors creating artefactual deaths (n = 99). Errors were corrected, resulting in retrospective changes of the official statistics.

The case-based retrospective inventory of the LTCF deaths conducted by AZG in Flanders led to a substantial update of the COVID-19 mortality database (Table 5). As a result of this inventory, 2,678 COVID-19 deaths (2,659 aggregated and 19 case-based deaths transmitted in the early stage of the epidemic by the RegHAs) were replaced by 2,557 case-based deaths.

**Table 5:** Changes following the inventory of COVID-19 deaths that occurred in LTCF, Flanders, 10 March–21 June 2020 (n = 2,678)

Outcome combining reporting methods	Number of LTCF		Number of COVID-19 deaths		Comparison of COVID-19 deaths by LTCF before and after the inventory			
			Initial registration	Inventory (case-based registration)				
					Over-reporting COVID-19 deaths		Missing COVID-19 deaths	
	n	%	n	n	n	%	n	%
LTCF reporting COVID-19 deaths with the initial registration (n = 415)								
Deaths in inventory	330	40.1	2,522 aggregated + 19 case-based	2,513	323	12.1	313	11.7
No death in inventory	79	9.6	122	0	122	4.6	NA	NA
No participation in inventory	6	0.7	15	15^a^	NA	NA	NA	NA
LTCF reporting no COVID-19 deaths with the initial registration (n = 407)								
Deaths in inventory	21	2.6	0	29	NA	NA	29	1.1
No death in inventory	386	47.0	0	0	NA	NA	NA	NA
Total	822^b^	100	2,678	2,557	445	16.6	342	12.8

COVID-19: coronavirus disease; NA: not applicable; LTCF: long-term care facilities.

^a^ The 15 deaths initially transmitted by the six NH that did not answer were kept in the updated database.

^b^ Including the eight NH situated in Brussels under the responsibility of the AZG.

Initially, 415 NH (50.4%) had reported those 2,678 COVID-19 deaths and 407 NH (49.6%) reported no COVID-19 deaths. Of the 415 NH that initially reported COVID-19 deaths, 330 also reported COVID-19 deaths in the inventory (n = 2,513), 79 reclassified COVID-19 deaths as non-COVID deaths (n = 122) and six did not answer, leaving 15 unconfirmed COVID-19 deaths that we have kept in the final database. Of the 407 NH that did not initially report COVID-19 deaths, 21 NH reported COVID-19 deaths (n = 29) only in the inventory. Furthermore, 386 NH consistently did not report any COVID-19 deaths in the aggregated registration, or in the inventory.

The final difference was the balance of many positive and negative changes at NH level. Of the 822 NH, 507 (61%) did not change their declaration, reporting either the same number of deaths or no deaths. Among the 330 NH reporting both aggregated and case-based COVID-19 deaths during the inventory, 119 NH reported the same number of deaths and 211 reported different death tolls in the aggregated and the case-based data. The aggregated data initially transmitted over-reported 323 deaths (12.1%) and missed 313 deaths (11.7%) (Table 5). In 90% of the 211 NH that reported a different number of COVID-19 deaths in aggregated and case-based ways, the changes were reasonably small, with differences ranging between − 6 and + 6 COVID-19 deaths over the period.

Beside those quantitative changes, the main added value of the inventory was to provide the details of deceased individuals; missing values about age and sex decreased from 98% to 0.5% of the deaths in the Flemish LTCF. 

After removing the excess deaths detected in all regions, the total toll decreased by 5% (370 excluded from Wallonia, Brussels and GSC regions + 121 excluded from Flanders/9,712 total deaths). However, this number concerns only detected errors and should therefore be considered as a minimum number of deaths counted in excess.

#### Reporting and dissemination

Between 14 March and 21 June 2020, COVID-19 mortality data were analysed and presented in 85 daily and 13 weekly epidemiological reports. During this period, there were 586,317 and 350,516 downloads of the daily report for the French and Dutch versions, respectively; the maximum number of downloads for each version (96,063 and 67,785, respectively) occurred during the week of 23 March, probably as awareness of the pandemic grew. COVID-19 mortality data were also included, among other indicators, in a special daily report for the Belgian Restricted Council of Ministers to inform them about the evolution of the epidemic. Detailed tabulations of the number of deaths broken down by all relevant variables were transmitted daily to the RegHAs.

## Discussion

This study describes the implementation of Belgium’s ad hoc emergency COVID-19 mortality surveillance. As a result of a rapid and full involvement of the hospitals and the NH, the establishment of comprehensive surveillance of COVID-19 deaths during the first epidemic wave was successful. Data received in real-time allowed a prompt assessment of the epidemiological situation, although a validation using death certificates will be necessary when possible.

In this ad hoc emergency surveillance, Belgium used broad criteria to define a COVID-19 death, which included deaths with any case-classification that occurred in any setting. We believe this decision was appropriate, based on several arguments. Firstly, since LTCF residents were not tested before the second half of April, attributing deaths to COVID-19 only in laboratory-confirmed cases, which accounted for 50% of the deaths, would have substantially underestimated the total number of COVID-19 deaths. This would have failed to raise or seriously delayed the severity of the outbreaks and the public and political awareness of the actual situation in NH. Comas-Herrera et al. previously stated that, when estimating the number of deaths in LTCF and particularly when initial testing priorities were entirely focused on hospitals, a system recording possible cases provided important and timely information on the scale of deaths linked to COVID-19 that can support decisions and is not subject to biases introduced by testing priorities [7]. Secondly, we found an excellent correlation between the daily number of COVID-19 deaths and the daily number of all-cause deaths during the first epidemic wave, using the Belgian Mortality Monitoring (Be-MOMO) [15,36], with a Spearman’s coefficient of 94% [12]. However, this correlation does not exclude the possibility of misclassified deaths, with the total excess deaths including both true COVID-19 deaths and excess deaths from other causes, as an indirect impact of COVID-19, e.g. not seeking medical advice or delaying treatment because of difficult access to hospitals or MDs. Moreover, the Belgian Geriatrics Society recommended avoiding hospitalisation of NH residents with a frailty score above 7, for fear that hospitals would be overwhelmed [37]. On the other hand, other common causes for deaths, e.g. road accidents, may have been prevented by the lockdown implemented as a measure to slow the spread of the virus during the first epidemic wave.

Belgium had one of the highest COVID-19 mortality rates worldwide. To what extent the high death count can be explained by the surveillance methodology is a valid question. It is well established that procedures for notifying COVID-19 deaths vary widely across countries [6,8]. The metadata of the deaths surveillance systems in numerous countries are documented by the Institut National d’Études Démographique’s COVID-19 portal [8], which also lists seven key issues affecting the countries’ comparability [38]: (i) the latency between occurrence of death and its publication, (ii) the death settings considered for inclusion, (iii) the criteria used to attribute the cause of death to COVID-19, including the availability of tests, (iv) the lag time between a calendar date and the start date of the epidemic, (v) retrospective updates affecting the dynamic of the daily death curve, (vi) the age and sex structure of the population, and (vii) the fact that differences in country size can lead to different geographical distributions of the burden, with large countries presenting important differences in the regional burden. Therefore, comparisons at national level can be misleading, and comparison of areas from similar size would be more relevant. However, the impact of country-specific methodological differences on the mortality rates has not yet been determined.

### Data quality

An ad hoc surveillance that was established rapidly under emergency circumstances to provide real-time data inevitably leads to certain errors. Misclassification by wrongly attributing deaths to COVID-19 is quite plausible in LTCF, as the diagnoses were mainly clinically based during the six first weeks of the epidemic, which probably resulted in a lack of specificity. Human errors in data entry included, among others, duplicates, errors in the personal details of deceased individuals, or the introduction of an incorrect number of deaths when providing aggregated data. The detection and resolution of obvious duplicates or errors in the personal details, e.g. incorrect date of birth in 2020, were addressed by automated programs. Suspicious errors, i.e. implausible values, warranting verification were put on a waiting list before entering the final database. This procedure limited the number of retrospective corrections. Very few checks could be carried out on the aggregated data. Replacing the aggregated data by case-based data for the Flemish NH through an inventory of the deaths solved this problem, but occurred only after the end of the first epidemic wave.

Errors detected after the initial data transmission resulted in delayed corrections in the final database. Although this is inherent to real-time monitoring, these corrections were sometimes misunderstood by data users, since consolidation is only possible after a few days or weeks. The number of corrections – pooled over all regions – that impacted the number of fatalities reached 5%, which is quite low, given the urgency under which the surveillance was established. Moreover, as the deaths that required most rectifications were distributed over the whole wave, they did not affect the conclusion and the management of the epidemic. However, this percentage should be considered a minimum estimate as it only reflects the detected errors.

An over-reporting of COVID-19 deaths occurred in some NH during the peak of mortality, between calendar weeks 13 and 16. This was most likely related to the combination of a large number of deaths and the lack of diagnostic tests; some errors were corrected afterwards by the NH. Over-reporting also occurred from errors in data entry. Even with case-based data, doubt remains that all data entry errors have been detected.

Although most NH have participated in data transmission at least once during the first epidemic wave, fewer reported COVID-19 deaths on a daily basis. It is therefore possible that COVID-19 deaths have been under-reported in NH. However, this seems unlikely, as the final database reflects that deaths of previous days were counted in the next reporting session with no decrease, e.g. on weekends.

Few deaths were reported in LTCF that were not NH, which was likely affected by several factors. The implementation of the surveillance in those settings is still in progress. The organisational landscape of non-NH LTCFs is more fragmented, making it more difficult to obtain exhaustive data from these institutions. The RegHAs also chose to focus mainly on NH where most deaths and cases occurred. Moreover, the small number of deaths in LTCF other than NH can also be explained by the fact that residents are younger and usually present fewer comorbidities than those living in NH.

Very few deaths were reported from the community via the MDs (0.6%). Deaths at home are most probably under-reported, but we currently have no information about the magnitude of this under-reporting. The excess in all-cause mortality has not been calculated by place of death, and death certificates were not yet available.

All 103 general hospitals participated in the SC survey, but not the psychiatric hospitals, who had to report to RegHAs. It is not known to what extent deaths that occurred in psychiatric hospitals were notified.

### Data flows, content and purpose of the surveillance data

The organisation of the data flow resulted in a very short latency for in-hospital deaths (95% of deaths notified with a latency of 1 day or less). The latency was slightly higher for NH, because of a longer data flow and the fact that not all NH reported every day. Large backlogs were mainly because of the broadening of the case definition and the retrospective inclusion of data from new sources.

The COVID-19 mortality surveillance in the 103 hospitals was relatively easy to implement owing to the SC survey, while implementation in more than 1,500 NH appeared to be more challenging. NH faced significant challenges and an overload of work because of the sudden onset of the epidemic, the lack of protective equipment, the shortage of staff to manage care for such a high number of patients. Moreover, NH had few experiences in participating in systematic disease registrations. Despite these difficulties, the implementation of a COVID-19 surveillance was set up quite early and was relatively complete. Belgium was fortunate to benefit from already existing public health networks and collaborations set up for different purposes, including surveillance and outbreak management of infectious diseases in NH [39]. In addition, during the last decade, several point prevalence studies on healthcare-associated infections and antimicrobial use have been conducted [40-42]; since the 2009/10 influenza season, a surveillance of influenza-like illness (ILI) has been implemented in Belgian NH [43]. Finally, in early 2020, an ILI surveillance project had been prepared and was ready to start but was paused when the COVID-19 epidemic began. It paved the way for COVID-19 surveillance in NH.

The COVID-19 mortality surveillance that was implemented in NH underscored the very high mortality among NH residents, which accounted for ca. two thirds of all COVID-19 deaths in Belgium during the first epidemic wave.

Nevertheless, the organisational complexity of health responsibilities in Belgium has made the death registration in NH a difficult task for Sciensano; the involvement of four RegHAs, each with their own tools and priorities, led to tedious work of harmonising all data into a single dataset after registration of deaths. This point should be kept in mind for improved efficiency in the future.

The monitoring of diseases and deaths can achieve multiple goals, each which might require different pieces of information depending on the objective [44]. The WHO recommends the registration of a minimal set of variables for each death, including age, sex, date of death and place of death [16]. In Belgium, at the start of the pandemic, different views coexisted among the actors within COVID-19 death surveillance about the format of data collection, i.e. whether it should be aggregated or case-based. For example, AZG argued that, at the height of the first epidemic wave, collecting aggregated total numbers per NH and per day was sufficient for operational purposes, e.g. detection of clusters in NH including the number of deceased individuals. They argued that the extra effort required by NH to collect individual death data could have had a deleterious effect on the response rate, which could have undermined the operational system in place. AZG therefore preferred to collect aggregated data, which was not broken down by age and sex during the emergency phase; they later carried out a case-based inventory of the deaths in NH after the peak of the epidemic. On the other hand, a notable drawback of aggregated data is a very limited possibility for data quality checks. Detailed information on sex, age, and date of death, (i) allows to measure age-specific case-fatality rate, (ii) to compare COVID-19 deaths with all-cause mortality by age group, (iii) to compare mortality rates in and outside NH after age-stratification [45], (iv) to allow the standardisation of mortality rates for international comparison and (v) for the development of predictive scenarios.

### Reporting and dissemination

The dissemination of the COVID-19 mortality data was of paramount importance during this epidemic. This data served as an indicator of the severity to inform policymakers on the epidemiological evolution and to guide the control measures accordingly. Moreover, it highlighted the precarious situation faced by many NH. The data was also used to inform the general population on the evolution of the epidemic. Of note, Flanders developed its own reporting system early in the first wave, and this could potentially explain the somewhat lower interest for downloading the Dutch versions of the national reports.

The dissemination of the mortality data was also useful for scientific efforts, in particular on the development of COVID-19 transmission models [46].

## Conclusions

We gained several valuable lessons learned through the establishment of COVID-19 mortality surveillance in Belgium. Firstly, establishing a surveillance in the LTCF was more challenging, but achieved through effective collaboration between the different levels of RegHAs, and facilitated by previous experience of surveys and surveillance activities in the sector. Secondly, the adoption of an extended definition of COVID-19 deaths in the framework of the surveillance, particularly in a context of limited testing capacity, provided timely information about the severity of the epidemic, and was validated by a good correlation with all-cause mortality. However, the diversity of registration tools decreased the efficiency of death reporting; the option of a common registration tool with language specifications should be considered. Thirdly, as rapid death registration in a crisis context can lead to registration errors, we recommend that the design of error detection methods and the implementation of error resolution protocols are elaborated within the different levels implied in the registration, and that a careful examination of the entered records is performed i.e. to check for duplicates, possible misclassification, outliers in the daily numbers of deaths, improbable values. Fourthly, we recommended the registration of six core variables for data validation and epidemiological surveillance: the date of birth, date of death, sex, case classification, place of death, and living in NH or not. Collection of these data proved to be feasible. Additional information on the postal code of the residence of deceased is valuable for geospatial analyses of the epidemic. However, data from real-time monitoring are provisional and require validation to ensure the quality of data for research. Finally, considering all the efforts made, in emergency, by all the actors in the COVID-19 mortality surveillance chain, it would be wise to consider in the future a system of electronic registration of death certificates in order to be able to collect information on causes of death in a timely manner. This would allow Belgium to better face future public health events with high mortality, to cover all places of death, to have case-based data, and not require disproportionate efforts from health and public health institutions to create or participate in ad hoc surveillance. At the end of the first wave, Belgium was better prepared for the registration of deaths for the subsequent waves of COVID-19.

## Supplementary Data

695000 Supplement
