# Supplementary material for: Establishing an ad hoc COVID-19 mortality surveillance during the first epidemic wave in Belgium, 1 March to 21 June 2020
Source: Euro Surveill. 2021 Dec 2;26(48):2001402. doi: 10.2807/1560-7917.ES.2021.26.48.2001402 (PMC8641068; doi:10.2807/1560-7917.ES.2021.26.48.2001402)
Supplement: Supplement [file 20-01402_SCOHY_Supplement.pdf]

Supplementary materials to "Establishing an ad hoc COVID-19 mortality surveillance during the first epidemic wave in Belgium, 1 March to 21 June 2020"

Renard F, Scohy A, Van der Heyden J, Peeters I, Dequeker S, Vandael E, Van Goethem N, Dubourg D, De Viron L, Kongs A, Hammami N, Devleesschauwer B, Sasse A, Rebolledo Gonzalez J, Bustos Sierra N.

"This supplementary material is hosted by Eurosurveillance as supporting information alongside the article [Establishing an ad hoc COVID-19 mortality surveillance during the first epidemic wave in Belgium, 1 March to 21 June 2020], on behalf of the authors, who remain responsible for the accuracy and appropriateness of the content. The same standards for ethics, copyright, attributions and permissions as for the article apply. Supplements are not edited by Eurosurveillance and the journal is not responsible for the maintenance of any links or email addresses provided therein."

Supplementary table 1 - Chronology of the evolution of the COVID-19 case definition

Date	Case definition
18/01/2020	Travelers coming back from Wuhan who develop respiratory symptoms during or within 14 days after traveling, should consult their medical doctor and mention the journey.
13/02/2020	• Travelers coming back from Wuhan who develop respiratory symptoms during or within 14 days after traveling, should consult their medical doctor and mention the journey.<br>• Persons who had contact with a lab-confirmed case in the 14 days prior to the onset of symptoms should also consult their physician and mention the contact.
18/02/2020	Every person with:<br>• upper/lower respiratory symptoms (acute start of minimal 1 of following symptoms: fever, cough, sore thought, myalgia, respiratory difficulties, headache,…)<br>AND<br>• or a travel history to China in the 14 days before the onset of the disease<br>• or a physical contact with a laboratory confirmed COVID-19 case in the 14 days prior to the onset of the disease.
26/02/2020	1. Every person with:<br>• upper/lower respiratory symptoms (acute start of minimal 1 of following symptoms: fever, cough, respiratory difficulties)<br>AND<br>• or a history of travel to a region of high transmission in the 14 days before the onset of the disease:<br>China, South-Korea, Iran and 11 municipalities in Italy (Codogno; Casapusterlengo; Castiglione d'Adda; Maleo; Fombio; Bertonico; Castelgerundo; Somaglia; San Fiorano; Terranova dei Passerini; Vo Euganeo)<br>• or physical contact with a laboratory confirmed case in the 14 days before the onset of the disease<br>2. Every person with:<br>• severe acute respiratory symptoms and/or clinical or radiological proof of pneumonia, needing hospitalisation

	AND<br>• History of travel to a region of high transmission in the 14 days before the onset of the disease: Other municipalities in Lombardia; Venet;, Emilia Romagna in Italy; Singapore; Japan.
28/02/2020	1. Every person with:<br>• Fever + clinical symptoms of a respiratory infection (cough, respiratory difficulties)<br>AND<br>• or a history of travel to region/country of high transmission in the 14 days before the onset of the disease:<br>China, South-Korea, Iran and Italy (provinces of Lombardia; Veneto and Emilia Romagna)<br>• or physical contact with a laboratory confirmed case in the 14 days before the onset of the disease<br>2. Every person with:<br>• severe acute respiratory symptoms and/or clinical or radiological proof of pneumonia, needing hospitalisation<br>AND<br>• History of travel to a region/country of high transmission in the 14 days before illness if the aetiological examination for endemic pathogens has remained negative: region/country mentioned above; Singapore; Japan.
04/03/2020	1. Every person with:<br>• Fever + clinical symptoms of a respiratory infection (cough, respiratory difficulties)<br>AND<br>• or a history of travel to region/country of high transmission in the 14 days before illness:<br>China, South-Korea, Iran and Italy (provinces of Lombardia; Veneto and Emilia Romagna)<br>• or physical contact with a laboratory confirmed case in the 14 days before illness<br>2. Every person with severe acute respiratory symptoms and/or clinical or radiological proof of pneumonia, needing hospitalisation if the etiological examination for endemic pathogens has remained negative.
05/03/2020	1. Every person with:<br>• Fever + clinical symptoms of a respiratory infection (cough, respiratory difficulties)<br>AND<br>• or a history of travel to a region/country of high transmission in the 14 days before illness:<br>China, South-Korea, Iran and North Italy (provinces of Lombardia; Veneto and Emilia Romagna; Piemonte; Ligurië; Trentino South-Tirol; Friuli-Venezia Giulia; Vlle d'Aosta and Marche)<br>• or physical contact with a laboratory confirmed case in the 14 days before illness<br>2. Every person with severe acute respiratory symptoms and/or clinical or radiological proof of pneumonia, needing hospitalisation after negative examination for bacterial infection

06/03/2020	1. Every person with:<br>• Fever + respiratory symptoms (cough and respiratory difficulties)<br>AND<br>• History of travel to a region of high transmission in the 14 days before illness: China, South-Korea, Iran and North Italy (provinces of Lombardia; Veneto and Emilia Romagna; Piemonte; Ligurië; Trentino South-Tirol; Friuli-Venezia Giulia; Vlle d'Aosta and Marche)<br><br>2. Every person with severe acute respiratory symptoms and/or clinical or radiological proof of pneumonia, needing hospitalisation after negative examination for bacterial infection
11/03/2020	Possible case:<br>Every person with acute upper/lower respiratory symptoms<br>- newly appearing<br>or<br>- deteriorating in case the patient suffers from chronic respiratory disease
04/04/2020	Possible case:<br>Every person with acute upper/lower respiratory symptoms<br>- newly appearing<br>or<br>- deteriorating in case the patient suffers from chronic respiratory disease<br><br>Radiologically confirmed case:<br>A radiologically confirmed case is a person in whom the laboratory test for COVID-19 is negative, but in whom the diagnosis of COVID-19 is made on the basis of a suggestive clinical presentation AND a compatible CT-scan of the chest.<br><br>Confirmed case:<br>Person with a lab confirmed COVID-19 infection
08/05/2020	Possible case:<br>A possible case of COVID-19 is a person with<br>- at least one of the following main symptoms: cough; dyspnoea; thoracic pain; acute anosmia or dysgeusia without obvious cause;<br>OR<br>- at least two of the following symptoms: fever; muscle pain; fatigue; rhinitis; sore throat; headache; anorexia; watery diarrhea with no apparent cause; acute confusion; sudden fall with no apparent cause ;<br>OR<br>- worsening of chronic respiratory symptoms (COPD, asthma, chronic cough...).<br><br>Radiologically confirmed case:<br>A radiologically confirmed case is a person in whom the laboratory test for COVID-19 is negative, but in whom the diagnosis of COVID-19 is made on the basis of a suggestive clinical presentation AND a compatible chest CT-scan.

15/05/2020	Confirmed case:<br>A confirmed case is defined as a person with laboratory confirmation of COVID-19 infection.<br>Possible case:<br>A possible case of COVID-19 is a person with<br>- at least one of the following main symptoms that appear acute, with no other obvious cause: cough; dyspnoea; thoracic pain; acute anosmia or dysgeusia;<br>OR<br>- at least 2 of the following symptoms, with no other obvious cause, fever; muscle pain; fatigue; rhinitis; sore throat; headache; anorexia; watery diarrhea; acute confusion; sudden fall ;<br>OR<br>- worsening of chronic respiratory symptoms (COPD, asthma, chronic cough...), with no other obvious cause.<br><br>Radiologically confirmed case:<br>A radiologically confirmed case is a person in whom the PCR for COVID-19 is negative, but in whom the diagnosis of COVID-19 is made on the basis of a suggestive clinical presentation AND a compatible chest CT scan.<br><br>Confirmed case:<br>A confirmed case is defined as a person where the diagnosis of COVID-19 infection has been confirmed by a molecular test .

Supplementary table 2 - Chronology of the COVID-19 deaths registration in nursing homes

Steps	Flanders	Wallonia	Brussels	German-speaking Community
First COVID-19 death in nursing homes	11/03/2020	16/03/2020	14/03/2020	27/03/2020
Registration of NH deaths by health inspectors, early steps of data centralization at Sciensano	11/03/2020	NA	11/03/2020	NA
Set up of an organized data exchange between Sciensano and regional authorities	17/03/2020 to 03/04/2020	17/03/2020 to 23/03/2020	17/03/2020 to 09/04/2020	NA
Online registration tool implemented, and start of the online registration	18/03/2020 Tool: e-loket (version 1) Aggregated data Place of death not available Data not transmitted (technical problem)	20/03/2020 Tool: Plasma Individual data	26/03/2020 Tool: LimeSurvey set up by Sciensano Aggregated data NB: in parallel, individual deaths details (health authorities)	26/03/2020 Tool: LimeSurvey set up by Sciensano Aggregated data
First data transmission to Sciensano via the online registration tool	1/4/2020 Not included immediately, since no information about place of death -> impossible to identify duplicates with the hospital surveillance	24/03/2020 Included in statistics	26/03/2020 Aggregated data not used, since individual data provided by health authorities	28/03/2020 Will be included on 22/4/2020 in the statistics
Change in case definition (Sciensano): retrospective inclusion in statistics of deaths of possible cases (outside hospital) Backlog (+85 deaths)	NA	30/03/2020	30/03/2020	NA

FLA: New version registration tool (e-loket) :	02/04/2020<br>e-loket<br>(version 2)<br>Place of deaths distinctable			
First inclusion in statistics of deaths data from the online tool	06/04/2020<br>Backlog<br>(+241 deaths)	24/03/2020<br>Included in statistics	10/04/2020<br>(before, individual data transmitted by health authorities)	21/04/2020<br>Backlog<br>(+22 deaths)
FLA: Investigation in NH to recover information on deaths having occurred in March 2020	09/04/2020<br>(backlog : +171 deaths)			
Start extensive testing in NH residents	15/04/2020	15/04/2020	15/04/2020	15/04/2020
Changes in registration of the diagnostic and registration of non-COVID-19 deaths	22/04/2020	15/04/2020	12/05/2020	12/05/2020
Individual data collected via the online registration tool (age, sex, date of death)	02/06/2020	20/03/2020	10/04/2020	12/05/2020
Replacement of previously aggregated data by case-based data	25/08/2020<br>(AZG inventory)			17/07/2020<br>(Sciensano investigation)

AZG: Agentschap Zorg en Gezondheid; NA: Not applicable

Supplementary table 3 – Variables collected by the different sources and transmitted to Sciensano, 2020

	Hospitals Belgium	LTCF Brussels	LTCF Wallonia	LTCF Flanders	LTCF German-speaking Community
**Date of birth (DOB)**	24/03 to 23/04 : Year of birth<br><br>Since 24/04 : DOB	Since start	Not available	Since 2/06 (earlier deaths updated on 25/08)	Since 12/05 (earlier deaths updated on 17/07)
**Age**	Calculated	Calculated	Since start	Calculated	Calculated
**Date of death (DOD)**	Since 24/03 from the surge capacity survey Since start from regional health authorities	Since start	Since start	18/3 to 1/6: proxy (updated on 25/08)<br><br>Since 2/06: exact DOD	28/03 to 12/05: proxy (updated on 17/07)<br><br>Since 12/05: exact DOD
**Sex**	Since 24/03	Since start	Since start	Since 2/06 (earlier deaths updated on 25/08)	Since 12/05 (earlier deaths updated on 17/07)
**Case-classification**	Since start: confirmed/ suspected<br><br>Since 11/04: + CT confirmed (previous deaths updated on 6/05)	Since start: confirmed/ possible<br><br>Since 12/05: + CT confirmed	Since start: confirmed/ possible<br><br>Since 12/05: + CT confirmed	Since start: confirmed/ possible	Since start: confirmed/ possible<br><br>Since 12/05: + CT confirmed
**Type place of death**	Hospital	Since start	Since start	Yes, since 2/04 (deaths 18/03 to 1/04 updated on 9/04)	Since start
**Type place of living**	Since 19/06	Since start Type of reporting LTCF as type of place of living	Since start Type of reporting LTCF as type of place of living	Since start Type of reporting LTCF as type of place of living	Since start Type of reporting LTCF as type of place of living
**Postcode place of death**	Since start	Since start	Since start	Since start	Since start
**Postcode place of residence**	Since 24/04	Since start Postcode of the LTCF reporting	Since start Postcode of the LTCF reporting	Since start Postcode of the LTCF reporting	Since start Postcode of the LTCF reporting

LTCF: Long-term care facilities
